# Supplementary figures and images for: Age-related changes in dermal collagen physical properties in human skin
Source: PLoS One. 2023 Dec 8;18(12):e0292791. doi: 10.1371/journal.pone.0292791 (PMC10707495; doi:10.1371/journal.pone.0292791)

Fig 3b original gel image

M      CTRL      hMMP-1      hMMP-1      CTRL      hMMP-1      CTRL  
X      X      X      X      \_\_\_\_\_      \_\_\_\_\_      X

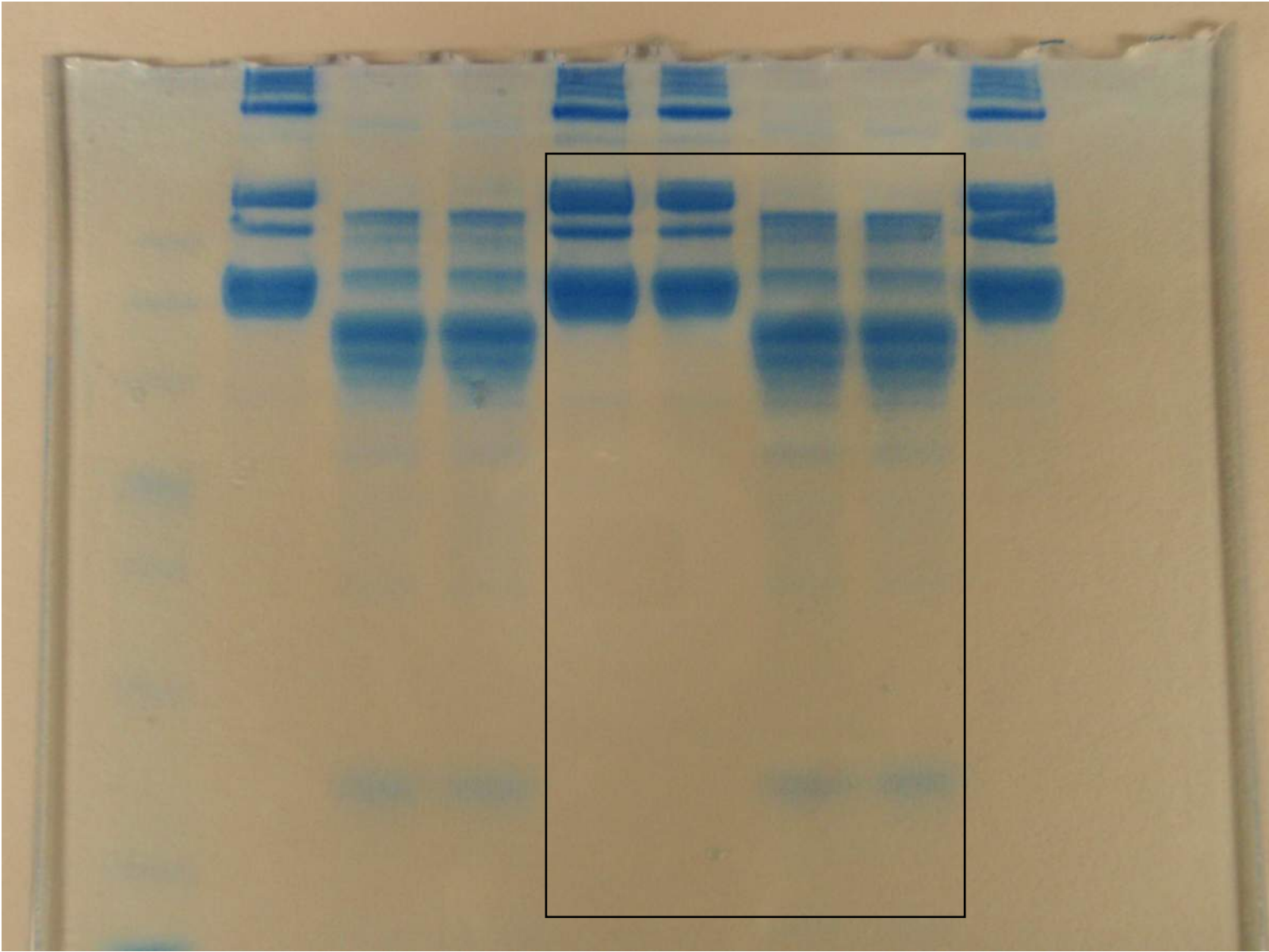

Supplement: S1 Raw image — (PDF) [file pone.0292791.s001.pdf]
