# Supplementary material for: Age-related changes in dermal collagen physical properties in human skin
Source: PLoS One. 2023 Dec 8;18(12):e0292791. doi: 10.1371/journal.pone.0292791 (PMC10707495; doi:10.1371/journal.pone.0292791)
Supplement: Attachment — Submitted filename: PlosOne letter for Reviewers.docx [file pone.0292791.s002.docx]

PONE-D-23-19389

TITLE: Age-related changes in dermal collagen physical properties in human skin

We appreciate the time and effort of the reviewers to provide their thoughtful comments regarding our manuscript.

In response to their suggestions, we conducted additional experiments (depicted in Fig 3cs and Fig 4a) and revised the manuscript.

Changes made to the manuscript are marked in the text.

Point-by-point responses to reviewers’ comments are below.

Reviewer #1:

Authors have drafted interesting piece of work which well aligned and well addressed.

Data of manuscript has been well articulated and hypothesis has well explained which fluently understandable by readers.

1. I recommend authors to go through entire manuscript once more for minor grammatical errors. I recommend to 1 change term Organ culture to Organotypic culture, to show the skin biopies were cultured and maintained under in vitro conditions.

*Response:* “Organ” culture changed to “Organotypic culture”, also corrected any grammatical errors.

2. Authors are recommended to replace old citation and cite recently published articles and to stick max 5 years old references only. (2018-2023).

*Response:* The citations have been updated (please refer to the "References" section), yet certain references remained unaltered due to the lack of more up-to-date sources.

Reviewer #2:

Authors tried to clarify the age-related changes in dermal collagen physical properties (surface roughness, stiffness, and hardness) using atomic force microscopy (AFM) and nanoindentation. They found that in the aged dermis, the surface of collagen fibrils was rougher, and fiber bundles were stiffer and harder, compared to young dermal collagen and that the age-related elevation of matrix metalloproteinase-1 (MMP-1) and advanced glycation end products (AGEs) might be responsible for rougher and stiffer/harder dermal collagen, respectively. These findings are interesting but there are some points to improve this paper.

Comment 1

Although some studies have already been carried out to clarify the age-related changes in dermal collagen physical properties by using atomic force microscopy (AFM) and nanoindentation, authors did not describe these works in the introduction, Authors should describe them and emphasize the importance of this work.

*Response:* By revisiting the Introduction section, we incorporated and deliberated upon the content of these papers (refer to the "Introduction" and “References” sections for more information).

Comment 2

In histology and morphometry (Page7), a scale of 1-9 for each parameter was used for the analysis. However, they did not describe the details of the difference of 1 to 9 scales. To understand the importance of Fig. 1b, they should show the differences of scales in thinness, space, and disorganization of dermal collagen fibers.

*Response:* We apologize for any confusion related to the scales utilized in our histological analysis. We have made revisions to the histological analysis scales and have provided a corresponding reference. Detailed information regarding these scales is available in the "Materials and Methods" section within the "Histomorphometry Analysis" category. Additionally, you can find supplementary information in the figure 1 legend.

Comment 3

In Figs 3 and 4, in addition to AFM data, skin section data should be necessary to be shown as the effects of MMP-1 digestion and ribose crossing on the dermis. Moreover, for Fig. 4, the presence of a glycated collagen matrix should be shown by histological immunostaining.

*Response:* Following the suggestions, we carried out additional experiments to verify the processes of MMP-1 induced collagen degradation (as shown in Fig 3c) and ribose-induced collagen glycation (depicted in Fig 4a). We would like to inform you that our endeavor to perform AEGs immunostaining was unsuccessful due to the issues with tissue freshness (new fresh skin tissue yielded positive results). In light of this, we quantified collagen glycation through fluorescence measurements (λex 370 nm/λem 440 nm). Further details regarding this methodology can be found in the "Materials and Methods" section of our study.

Comment 4

In the paper, published in International Journal of Nanomedicine 2017:12 3303–3314, “Combining nano-physical and computational investigations to understand the nature of “aging” in dermal collagen”, an age-related decrease in the Young’s modulus of the transverse fibril (from 8.11 to 4.19 GPa in young to old volunteers, respectively, P,0.001) was reported. Authors should discuss the discrepancy.

*Response:* We referenced and discussed above paper (see “Discission” for details).

Comment 5

In Figs 3c and 4c, the spelling of punch in “Young skin puch biopsy” should be corrected.

*Response:* The typos are corrected.
